# NIPK, a protein pseudokinase that interacts with the C subunit of the transcription factor NF-Y, is involved in rhizobial infection and nodule organogenesis

**DOI:** 10.3389/fpls.2022.992543

**Published:** 2022-09-21

**Authors:** Joaquín Clúa, Carolina Rípodas, Carla Roda, Marina E. Battaglia, María Eugenia Zanetti, Flavio Antonio Blanco

**Affiliations:** Instituto de Biotecnología y Biología Molecular, Facultad de Ciencias Exactas, Universidad Nacional de La Plata, CCT-La Plata, CONICET, La Plata, Argentina

**Keywords:** protein kinase, nitrogen fixation, signaling, symbiosis, transcription factor

## Abstract

Heterotrimeric Nuclear Factor Y (NF-Y) transcription factors are key regulators of the symbiotic program that controls rhizobial infection and nodule organogenesis. Using a yeast two-hybrid screening, we identified a putative protein kinase of *Phaseolus vulgaris* that interacts with the C subunit of the NF-Y complex. Physical interaction between NF-YC1 Interacting Protein Kinase (NIPK) and NF-YC1 occurs in the cytoplasm and the plasma membrane. Only one of the three canonical amino acids predicted to be required for catalytic activity is conserved in NIPK and its putative homologs from lycophytes to angiosperms, indicating that NIPK is an evolutionary conserved pseudokinase. Post-transcriptional silencing on *NIPK* affected infection and nodule organogenesis, suggesting NIPK is a positive regulator of the NF-Y transcriptional complex. In addition, *NIPK* is required for activation of cell cycle genes and early symbiotic genes in response to rhizobia, including *NF-YA1* and *NF-YC1*. However, strain preference in co-inoculation experiments was not affected by *NIPK* silencing, suggesting that some functions of the NF-Y complex are independent of NIPK. Our work adds a new component associated with the NF-Y transcriptional regulators in the context of nitrogen-fixing symbiosis.

## Introduction

Nitrogen scarcity in soils is a major constraint for plant growth (Mueller et al., 2012). This limitation has been overcome by the exogenous application of inorganic fertilizers, causing negative effects on the environment and human health. One of the biggest challenges of modern agriculture is improving crop yields and, at the same time, reducing the negative consequences of the crop management introduced during the green revolution (Bailey-Serres et al., 2019). Symbiotic association of plants with beneficial microorganisms has a positive impact on nutrient uptake, allowing roots to expand their capacity to incorporate nitrogen and phosphorus from soils. Optimization of these symbiotic associations would greatly contribute to sustainability of agricultural practices. The best characterized symbiotic interaction between plants and microorganisms is the association between legumes and diazotrophic bacteria known as rhizobia. This interaction is initiated by an exchange of signals between both symbionts and the triggering of a signal transduction pathway in the plant that activates two genetic programs that are tightly coordinated, the rhizobium infection and the organogenesis of the nodule (Oldroyd et al., 2011). The infection process allows rhizobia to penetrate root tissue and reach the cortical cells that will form the nodule. In most economically important legumes, infection proceeds by the formation of a plant-derived hollow structure called the infection thread (IT), which is initiated at the tip of the root hair and grows inward to reach the cortical cells that have reinitiated cell divisions to form the nodule primordia (Roy et al., 2020).

The two genetic programs associated with the root-nodule symbiosis are under the control of several transcription factors, including Nodule Inception (NIN; Schauser et al., 1999), ERF Required for nodulation (ERN1 and ERN2; Middleton et al., 2007; Cerri et al., 2012), Nodulation Signaling Pathway 1 and 2 (NSP1 and NSP2; Kalo et al., 2005; Smit et al., 2005), CYCLOPS (Messinese et al., 2007; Yano et al., 2008; Horváth et al., 2011), and members of the heterotrimeric Nuclear Factors Y (NF-Y) family (Combier et al., 2006; Zanetti et al., 2010; Laporte et al., 2013; Soyano et al., 2013). NF-Y transcription factors, which are composed by three subunits named NF-YA, NF-YB, and NF-YC, bind with high affinity to the CCAAT box sequences present in eukaryotic promoters, promoting transcriptional activation of their target genes (Dolfini et al., 2012; Zanetti et al., 2017). Molecular and genetic studies have shown strong evidence of the connection of NF-Y family members with other transcription factors to exert a central role of NF-Ys in the transcriptional responses during symbiosis. *LjNF-YA1* and *LjNF-YB1* are under the control of NIN in *Lotus japonicus* (Soyano et al., 2013). Consistently, *MtNF-YA1* is modulated by NIN in *Medicago truncatula* (Laloum et al., 2014). It has also been described that the complex formed by MtNF-YA1, MtNF-YC2, and MtNF-YB16 recognizes CCAAT elements in the *ERN1* promoter, activating its expression (Laloum et al., 2014; Baudin et al., 2015). Genetic and functional studies revealed that *MtNF-YA1* is required for persistence of the meristem of indeterminate nodules, where its spatial expression is regulated by microRNA 169a (miRNA169a; Combier et al., 2006). Similarly, silencing of *LjNF-YA1* also produces defects in the organogenesis of determinate nodules (even though they do not have a persistent meristem), since LjNF-YA1 regulates cell division of cortical cells through the activation of Cyclin B1 (Soyano et al., 2013). Later on, it was shown that *MtNF-YA1* is also required for infection thread progression (Laloum et al., 2014). In addition to cell cycle genes, NF-Ys control the expression of genes that encode transcriptional regulators of the SHORT INTERNODES/STYLISH family and their downstream targets *YUCCA1* and *YUCCA11* (Hossain et al., 2016; Shrestha et al., 2021).

In common bean (*Phaseolus vulgaris*), the NF-YA1 and NF-YC1 subunits also control infection and nodule organogenesis and, in addition, the selective response of the plant that leads to the selection of rhizobial strains that have coevolved with the Mesoamerican accession at this diversification center (Zanetti et al., 2010). The number of nodules is affected by the knockdown of *PvNF-YA1* or *PvNF-YC1*, which, in turn, control the expression of cell cycle genes that regulate the G2/M transition (Zanetti et al., 2010; Rípodas et al., 2019). In addition, *NF-YC1*, *NF-YB7*, and *NF-YA1* are part of the strain-preference mechanism that is present in Mesoamerican cultivars, where strains of *Rhizobium etli* carrying the α allele of the *nodC* gene (*nodC*-α) form more nodules than strains with the *nodC*-δ allele in co-inoculation experiments. All these evidence support a central regulatory role of NF-Y transcription factors in different aspects of the response of legumes to their cognate symbionts.

NF-Y subunits can interact with other transcription factors to form transcriptional regulatory complexes different than the canonical heterotrimer in different plant species (Masiero et al., 2002; Yamamoto et al., 2009; Liu and Howell, 2010; Hwang et al., 2019). Using the yeast two-hybrid system, we previously reported that NF-YC1 interacts with SIN1 (Scarecrow-like13 Involved in Nodulation), a transcription factor of the GRAS family (Battaglia et al., 2014). *SIN1* is involved in rhizobial infection and nodule organogenesis, as well as in lateral root development (Battaglia et al., 2014). As in the case of NF-YA1 and NF-YC1, SIN1 controls the transcriptional activation of G2/M transition cell cycle genes (Battaglia et al., 2014). Here, we report another NF-YC interacting protein detected in the same yeast two-hybrid screening, a protein kinase designated as NF-YC1 Interacting Protein Kinase (NIPK). NF-YC and NIPK interaction occurs in the cytoplasm and NIPK is required for rhizobial infection and nodule organogenesis, but not for the selection of the rhizobia strain that will occupy nodules in common bean.

## Materials and methods

### Biological material and plant transformation

Plant growth and transformation were performed as previously described (Blanco et al., 2009; Zanetti et al., 2010). *Rhizobium etli* strains SC15 and 55 N1, as well as the strain CFNx5 expressing the DsRed, were previously reported (Aguilar et al., 2004; Smit et al., 2005).

### Yeast two-hybrid assay

Diploid yeasts carrying the complete open reading frame of *NIPK* in the pGADT7 vector (Clontech) and different versions of *NF-YC1* in pGBKT7 were generated by mating of haploid Y187 and AH109 strains (Clontech). Yeasts were incubated at 28°C for 2 days in liquid media and then 5 μl were spotted and cultivated in solid Synthetic Defined (SD) media complemented with Double Dropout (DDO, without Leu and Trp), Triple Dropout (TDO, without Leu, Trp and Ade) or Quadruple Dropout (QDO, without Leu, Trp, His and Ade). The SD-TDO and QDO media were supplemented with 5 mM 3-amino-1,2,4 triazole. Positive and negative controls, provided with the kit, were p53 interacting with AgT and LamC, respectively (Clontech). β-galactosidase activity was measured using ortho-nitrophenyl-β-galactoside as substrate, following the protocol supplied by Clontech (Yeast Protocols Handbook). One unit of β-galactosidase activity was defined as the amount of enzyme that hydrolyzes 1 μmol of ONPG to o-nitrophenol and D-galactose per min per cell (Miller unit).

### Plasmid construction

To create constructs for RNAi 1 and RNAi 2 of *NIPK*, PCR fragments of 100 bp of the 3′ UTR region and 311 bp corresponding to the kinase domain were obtained using primers NIPK RNAi 1 and 2 (Supplementary Table S1), respectively, and cDNA of *P. vulgaris* as template. DNA fragments were cloned in pENTR/D-TOPO (Invitrogen) and recombined in pK7GWIWG2D(II; Karimi et al., 2002). The control vector *GUS* RNAi was previously obtained in the laboratory (Blanco et al., 2009).

For subcellular localization, the region corresponding to the ORF of *NIPK* was amplified with primers NIPK ORF (Supplementary Table S1), cloned into pENTR/D-TOPO, and recombined into the GATEWAY compatible vector pMDC43 (Curtis and Grossniklaus, 2003).

For Bimolecular Fluorescence Complementation (BiFC) assays, the ORFs of *NF-YC1* and *NIPK* were amplified with M13 primers from the corresponding pENTR/D-TOPO vectors and the resulting fragments were then recombined into the GATEWAY compatible vectors pGPTVII.Bar.YN-GW and pGPTVII.Hyg.GW-YC (Hirsch et al., 2009), respectively.

### Subcellular localization, bimolecular fluorescence complementation, and co-immunoprecipitation assays

*Agrobacterium tumefaciens* strain GV3101 was transformed with the constructs for localization and bimolecular fluorescence complementation (BiFC). Agroinfiltration was performed as previously described (Voinnet et al., 2003; Battaglia et al., 2014). Strains carrying each construct were combined adding equal volumes before agroinfiltration. Plasmolysis was carried out by incubating leaf sections with 30% (v/v) glycerol before microscopic examination of tissue. Leaves were observed 2–3 days after agroinfiltration in a Leica SP5 confocal microscope.

Tissue for co-immunoprecipitation assays was collected at 3 days after agroinfiltration and total proteins extracted in 10 mM Tris pH 7.5, 150 mM NaCl, 5 mM DTT, 10% (v/v) glycerol, 1 mM EDTA, 0.1% (v/v) Triton X-100, 5 mM CaCl_2_, 2% (p/v) PVPP and 1.65% (v/v) of protease inhibitors (Sigma-Aldrich). Samples were incubated for 30 min at 4°C with agitation and then centrifuged at 2,400 *g* for 15 min at 4°C. Forty microliter of anti-FLAG conjugated to agarose beads (Sigma-Aldrich) was added to the supernatant and incubated in a rocking shaker for 2 h at 4°C. After centrifuging at 17,000 *g*, the precipitates were washed six times with 1 ml of extraction buffer without PVPP and incubated in elution buffer (200 ng/μl of Sigma-Aldrich 3X FLAG peptide in extraction buffer without PVPP) for 10 min at 4°C. The supernatant was recovered and subjected to Western blot analyses using anti-FLAG (1:500; Sigma-Aldrich) or anti-GFP (1:5,000; Invitrogen) antibodies.

### Phenotypic analyses

Composite plants were generated as described (Blanco et al., 2009). Roots that did not express GFP were removed before inoculation. Nodules were counted in individual roots at 7, 14, and 21 days post-inoculation (dpi). Nodules were photographed at 21 dpi and nodule diameter was measured using ImageJ. Infections were analyzed in roots inoculated with a *R. etli* strain CFNX5 that expressed DsRed. Four days after inoculation, ITs were visualized in an inverted IX51 Olympus microscope, quantified, and classified in those that were in the root hair (Root hair, RH), reached the base of the trichoblast (Epidermis, EP), or progressed to cortical tissue (Cortex, CX) as previously described (Battaglia et al., 2014). All experiments were performed in biological triplicates. Optical microscopy of nodule sections was performed as previously described (Zanetti et al., 2010). For strain preference analysis, roots were inoculated with a 1:1 mix of *R. etli* SC15 and 55 N1 strains. Nodules collected at 21 dpi were sterilized with 96% (v/v) ethanol for 30 s and 6 min in 9% (v/v) hydrogen peroxide, washed 6 times with distilled water, and crushed individually in ELISA plates containing 5 μl of water. The suspension was transferred to Petri dishes containing YEM media supplemented with Congo red as described (Aguilar et al., 2004). Polymorphism of the *nodC* gene was determined by RAFLP as previously reported (Aguilar et al., 2004).

### RT-qPCR

RNA extraction, cDNA synthesis, and qPCR experiments were performed as described (Peltzer Meschini et al., 2008). Primers for *NIPK* and *Phvul011G070500*, *CYCB*, *CDC2*, *ERN1*, *ENOD40*, *PvNF-YA1*, *PvNF-YC1*, and *EF1-α* are listed in Supplementary Table S1.

## Results

### *NIPK* encodes a pseudokinase that interacts with NF-YC1 in a yeast two-hybrid screening

**Figure 1:**
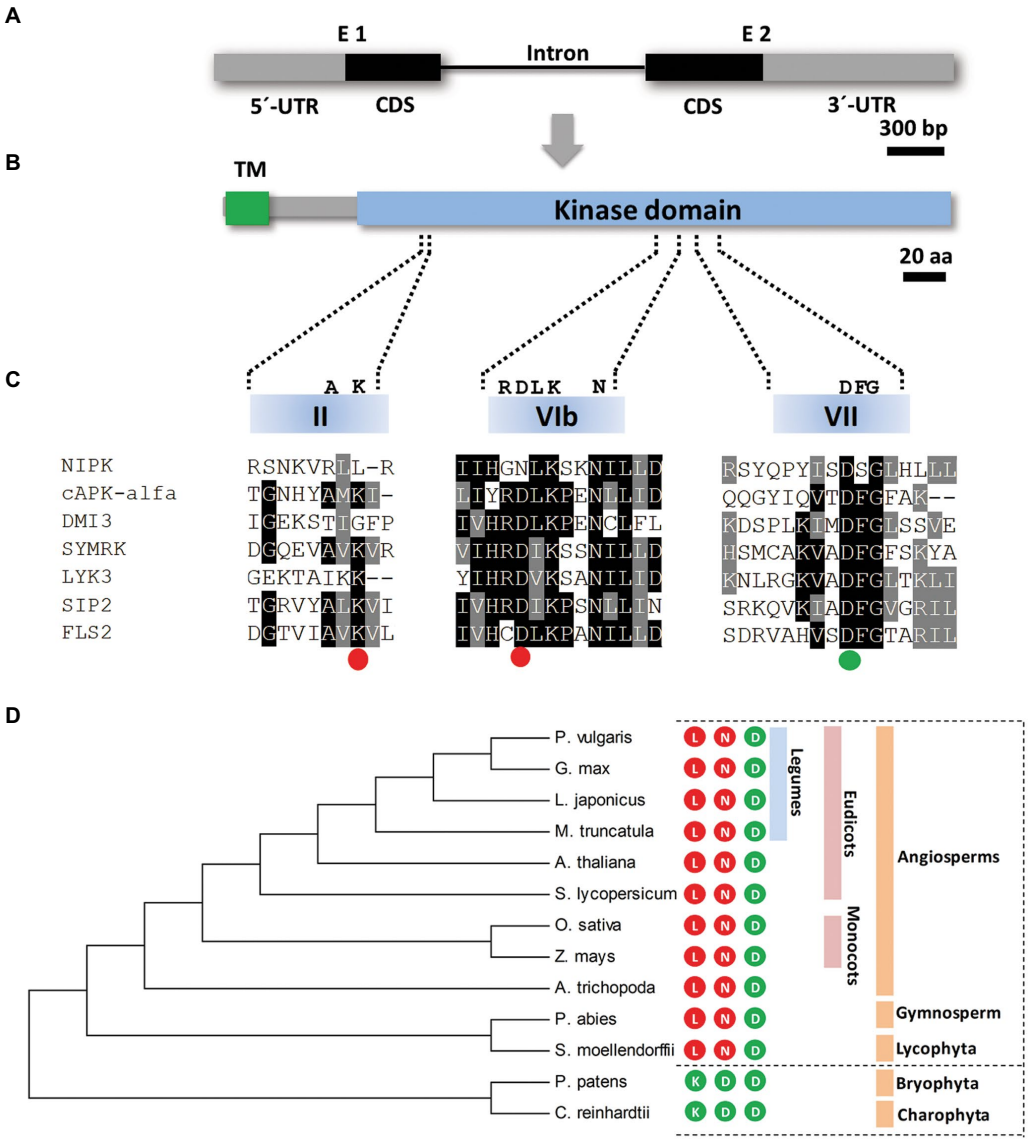
*NIPK* encodes a protein pseudokinase. **(A,B)** Schematic representation of the *NIPK* gene **(A)** and the encoded protein **(B)**. Gray boxes correspond to 5′ and 3′ untranslated regions (UTR) and black boxes correspond to coding sequence regions. The black line indicates the only intron present in the *NIPK* gene. The putative transmembrane (TM) and the kinase domains of the protein are shown in green and light blue, respectively. **(C)** A multiple sequence alignment of motives VAIK (II subdomain), HRD (VIb subdomain), and DFG (VII subdomain) of the kinase domain of NIPK and the functional kinases DMI3 (Gleason et al., 2006), SYMRK (Yoshida and Parniske, 2005), LIK3 (Jayaraman et al., 2017), SIP2 (Chen et al., 2012), FLS2 (Lu et al., 2010), and cPKA-alfa. The colored circles indicate whether each of the three amino acids (K, D, and D) required for the phosphotransfer reaction are conserved (green) or not (red) in NIPK. **(D)** Phylogenetic tree generated with the amino acidic sequences of *P. vulgaris* NIPK and its putative orthologs from *Medicago truncatula*, *Arabidopsis thaliana*, *Lotus japonicus*, *Glycine max*, *Amborella trichopoda*, *Solanum lycopersicum*, *Zea mays*, *Oryza sativa, Selaginella moellendorffii, Picea abies, Physcomitrella patens*, and *Chlamydomonas reinhardtii*. The phylogenetic tree was generated using MEGA7. Numbers represent bootstrap values obtained from 1,000 trials. The colored circles indicate whether each of the three amino acids required for the phosphotransfer reaction (K, D, and D) are present (green) or not (red) in the amino acid sequence of NIPK orthologs in each species.

In order to identify proteins that potentially interact with NF-YC1, in a previous work, we reported a yeast two-hybrid screening using NF-YC1 as a bait and a common bean cDNA library (Battaglia et al., 2014). Forty-five positive clones corresponding to eight different cDNAs were obtained. One of these cDNA clones corresponds to a *P. vulgaris* gene (*Phvul.011G181900*) that contains a coding sequence of 1,062 bp, interrupted by a single intron of 1,035 bp and flanked by 5′- and 3′-untranslated regions (UTR) of 677 and 980 bp, respectively (Figure 1A). The predicted protein contains 353 amino acids with a putative transmembrane domain (TM) at the N-terminus and a kinase domain of 285 amino acids at the C-terminus according to the TMHMM^1^ and UniProt^2^ analyses (Figure 1B). Based on these results, we named the *Phvul.011G181900* gene *NIPK*, for NF-YC1 Interacting Protein Kinase. A multiple sequence alignment revealed the presence of the 11 characteristic regions of the kinase domain (Hanks et al., 1988; Supplementary Figure S1). Nevertheless, two of the three amino acids considered to be indispensable for the kinase catalytic activity (Hanks, 2003), are not conserved in the NIPK amino acid sequence (Figure 1C; Supplementary Figure S1). The lysine residue in subdomain II that is required to anchor and orient ATP and the aspartic acid of subdomain VIb that functions as the catalytic base in the phosphotransfer reaction (Hanks, 2003) are replaced by a leucine and an asparagine in NIPK, respectively. Only the aspartic acid of subdomain VII, required to anchor and orient ATP, is conserved in NIPK. The three amino acids in these particular positions are conserved in the *NIPK* putative orthologous genes from other plant species (Figure 1D; Supplementary Figure S2), including lycophytes, gymnosperms, and angiosperms. Taken together, this analysis suggests that NIPK is, based on its sequence, an evolutionary conserved pseudokinase, i.e., a protein kinase predicted to be catalytically inactive (Boudeau et al., 2006).

### NIPK interacts with NF-YC1 in yeast and *in planta*

**Figure 2:**
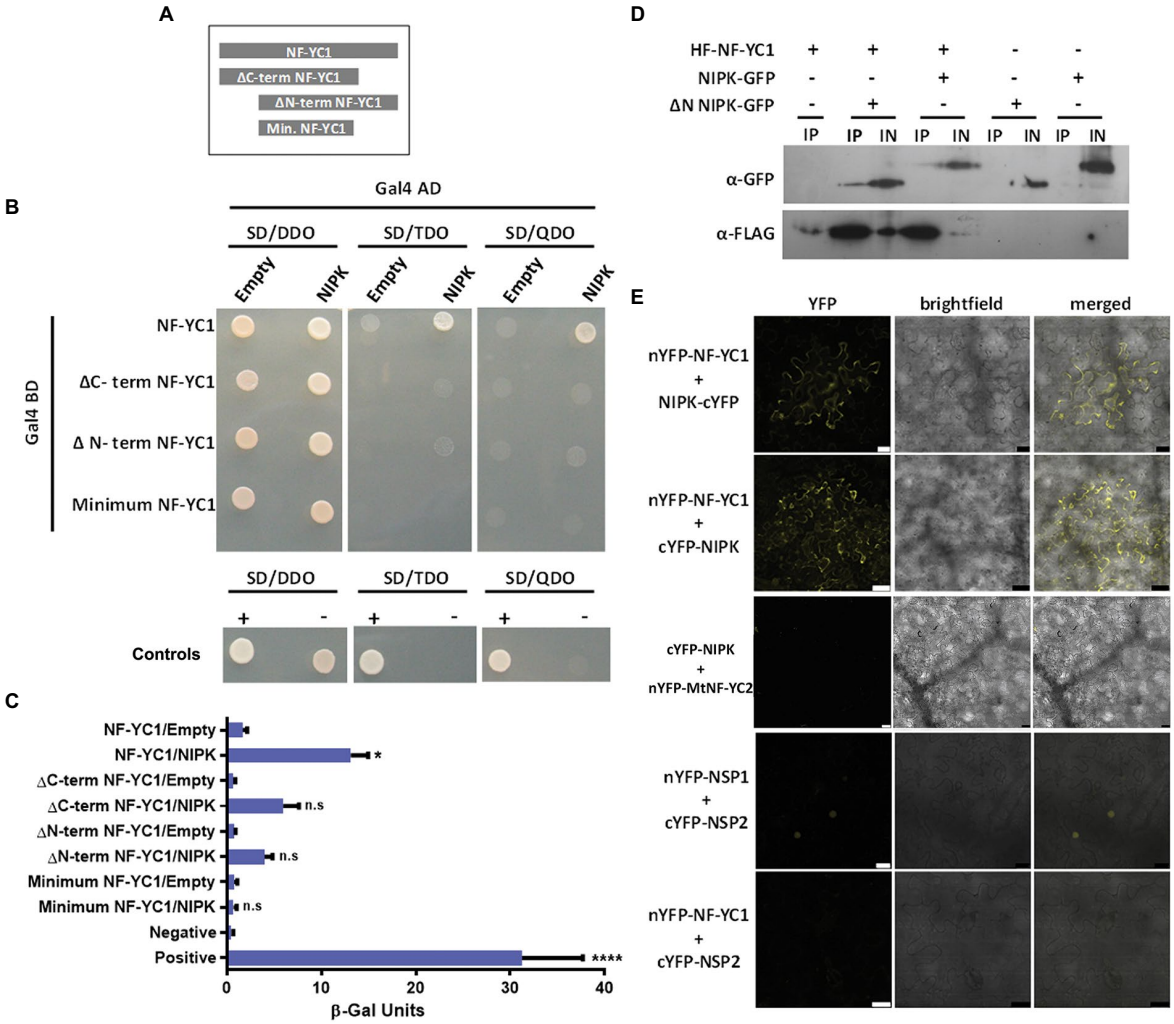
NIPK interacts with NF-YC1 in yeast and in planta. **(A)** Scheme of NF-YC1 versions used as baits in a yeast two-hybrid assay. **(B)** Interaction in yeast using the two-hybrid system. The pGBKT7 plasmid containing the Gal4 BD was fused to *NF-YC1* or different truncated versions indicated in panel **(A)** and introduced into the Y187 strain. The pGADT7 plasmid containing the activation domain of Gal4 (AD) was fused to *NIPK* and introduced into the AH109 strain. The Y187 strains carrying the different versions of NF-YC1 were mated with the strain AH109 carrying the NIPK AD fusion or the empty vector. Strains were selected in synthetic defined media (SD) lacking leucine and tryptophan (double dropout, SD-DDO); leucine, tryptophan, and adenine (triple dropout, SD-TDO) or leucine, tryptophan, adenine, and histidine (quadruple dropout, SD-QDO). Positive and negative controls are p53 interacting with AgT and LamC, respectively. **(C)** The interaction was quantified using a β-Galactosidase assay with ONPG as substrate. Bars represent the media and SEM of three independent experiments. Asterisks indicate significant differences with the control in an ANOVA test followed by a Tukey test for multiple comparisons (*, *p* < 0.05; ****, *p* < 0.0001; ns, not significant). **(D)** Co-immunoprecipitation assay. His-FLAG tagged NF-YC1 (HF-NF-YC1) was co-expressed with the GFP tagged NIPK (NIPK-GFP) or a truncated version lacking the N-terminus transmembrane domain (GFP-∆N NIPK) in *Nicotiana benthamiana* leaves. The crude extract or input (IN) and the fraction immunoprecipitated with FLAG antibodies (IP) were analyzed by Western blot using anti-GPF or anti-FLAG antibodies (α-GFP and α-FLAG). **(E)** Bimolecular fluorescence complementation assay. *Nicotiana benthamiana* leaves were co-infiltrated with nYFP-NF-YC1 in combination with cYFP-NIPK, NIPK-cYFP, or cYFP-NSP2 (negative control). The interaction between *Medicago truncatula* NSP1 and NSP2 is shown as a positive control. Confocal laser microscopy images of the YFP fluorescence (left panels), bright field (middle panel), and merged (right panel) are shown. Scale bars: 50 μm.

To verify the interaction between NIPK and NF-YC1, we conducted a yeast two-hybrid assay with the full-length ORFs of both proteins. Expression of translational fusions of *NF-YC1* with the *GAL4* binding domain (BD-NF-YC1) and *NIPK* with the *GAL4* activation domain (AD-NIPK) allowed yeast growth under high stringent selection conditions, whereas no growth was observed when BD-NF-YC1 was co-expressed with the empty vector (Figures 2A,B). This observation was confirmed and quantified in a β-galactosidase activity assay (Figure 2C). Deletion of either the C- or N-terminus of NF-YC1 strongly compromised its interaction with NIPK (Figures 2B,C), indicating that the central region is not sufficient for the interaction with NIPK in yeast. The N- and C-terminal have shown to be required for DNA binding and the interaction of NF-YC with the other two subunits of the functional NF-Y trimer (Romier et al., 2003), whereas a region with homology to histones, named histone fold domain, encompasses the central part of the protein (Laloum et al., 2013). Our results indicate that the regions flanking the histone fold domain of NF-YC1 are required for the interaction with NIPK in the yeast two-hybrid system.

To test the interaction *in planta*, we performed co-immunoprecipitation (CoIP) and bimolecular fluorescence complementation (BiFC) experiments in *Nicotiana benthamiana* leaves. For the former, we co-expressed a His-FLAG (HF) tagged version of NF-YC1 together with a GFP-tagged NIPK (NIPK-GFP) or a truncated version of NIPK lacking the N-terminal region located upstream of the kinase domain (GFP-∆N NIPK), which does not contain the TM domain. Immunoprecipitations were performed using anti-FLAG antibodies and the presence of NIPK was detected by immunoblot using anti-GFP antibodies (Figure 2D). The results show that NIPK physically interacts with NF-YC1 *in planta* and that the N-terminal region of NIPK is not essential for the interaction. To perform the BiFC experiment, we co-expressed the split N-terminus of YFP (nYFP) fused to NF-YC1 and the C-terminus of YFP (cYFP) fused to either the N- or C-terminus of NIPK. Two days after co-agroinfiltration of *N. benthamiana* leaves, a fluorescent signal corresponding to the wavelength of YFP emission was detected in the cytoplasm of epidermal cells, whereas no signal was detected when nYFP-NF-YC1 was co-expressed with cYFP-NSP2 (Figure 2E). A strong fluorescence signal in the nucleus was visualized when NSP1 and NSP2 from *M. truncatula* were used as a positive control of a BiFC interaction (Hirsch et al., 2009). In summary, these results suggest that NIPK specifically interacts with NF-YC1 both in yeast and *in planta*, and that the interaction occurs in the cytoplasm of *N. benthamiana* epidermal leaves cells. Whereas the kinase domain of NIPK is sufficient for heterodimer formation, deletion of either the N- or C-terminus of NF-YC1 compromised the interaction.

### NIPK localizes at the cytoplasm and the plasma membrane of *Nicotiana benthamiana* cells

**Figure 3:**
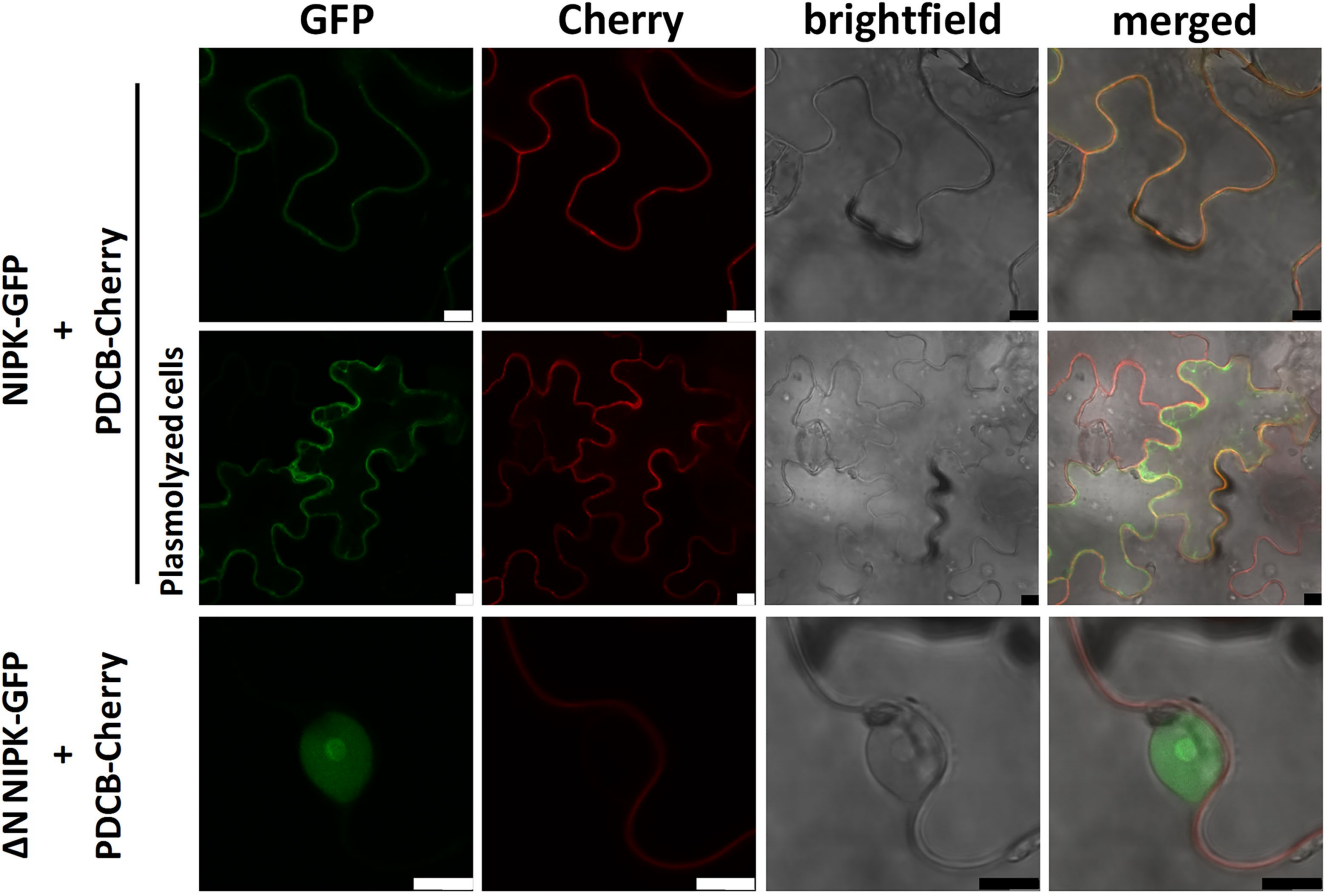
Subcellular localization of NIPK. *Nicotiana benthamiana* epidermal leaf cells co-expressing NIPK-GFP together with PDCB-Cherry as a plasma membrane marker in non-plasmolyzed or plasmolyzed cells (upper and middle panels, respectively). Lower panels show the subcellular localization of GFP-∆N NIPK and the plasma membrane marker in non-plasmolyzed cells. Confocal laser microscopy images of the GFP and Cherry fluorescence, bright field, and merged are shown. Scale bar: 10 μm.

Since NF-YC1 was shown to be distributed between the nucleus and the cytoplasm (Zanetti et al., 2010) and NIPK interacts with NF-YC1 in the cytoplasm, we explored the subcellular localization of NIPK. NIPK fused to GFP was co-expressed with the plasma membrane marker *plasmodesmata callose-binding protein 1* (PDCB) fused to Cherry (Simpson et al., 2009). Confocal laser microscopy of agroinfiltrated *N. benthamiana* epidermal cells revealed that NIPK-GFP co-localized with PDCB-Cherry at the plasma membrane (Figure 3, upper panels). However, after cells were plasmolyzed, NIPK-GFP was also visible in the cytoplasm, whereas PDCB-Cherry was visible only in the plasma membrane (Figure 3, middle panels). These results suggest that NIPK localizes at both the cytoplasm and the plasma membrane. In order to analyze if the putative N-terminus transmembrane domain of NIPK is required for the observed localization, we constructed a truncated version of NIPK lacking this domain fused to GFP (GFP-∆N NIPK). Two days after agroinfiltration, GFP-∆N NIPK localized at the nucleus and nucleolus (Figure 3, lower panels), suggesting that the transmembrane domain was determinant for the localization of NIPK at the plasma membrane and the cytoplasm. To further characterize the subcellular localization of NIPK in the context of the dimerization with NF-YC1, we transiently co-expressed the translational fusions NIPK-GFP and HF-NF-YC1, or NIPK-HF and NF-YC1-GFP in *N. benthamiana* epidermal cells. The results showed that overexpression of one of the interacting partners did not significantly change the subcellular localization of the other with respect to controls (Supplementary Figure S3), suggesting that NF-YC1 dissociates from NIPK before its translocation to the nucleus.

### *NIPK* is induced in roots by rhizobia

**Figure 4:**
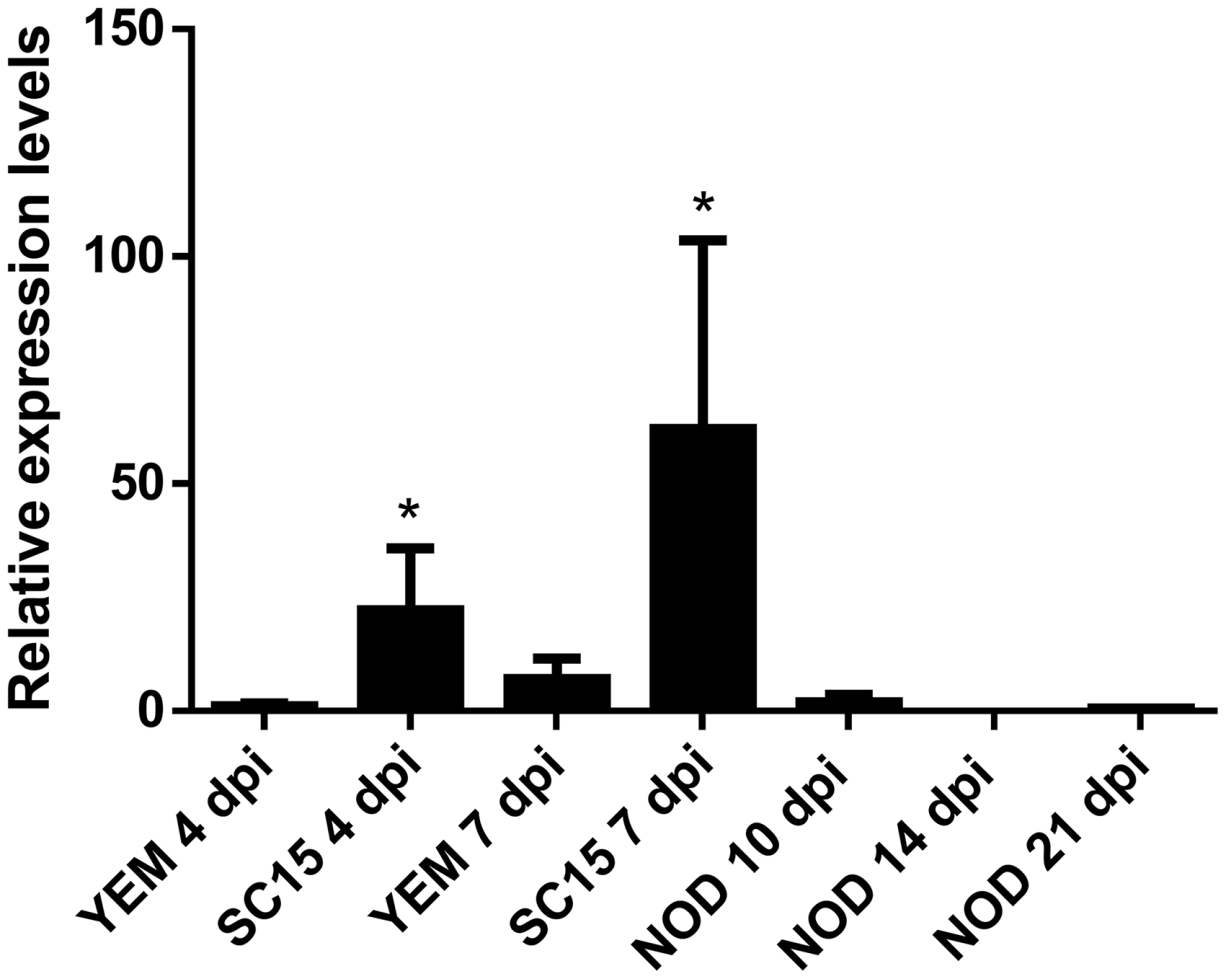
Expression of *NIPK* in response to rhizobia. RT-qPCR analysis showing relative expression of *NIPK* in roots 4 and 7 days after infection with the strain SC15 of *Rhizobium etli* or mock-inoculated roots (YEM) or nodules collected at 10, 14, and 21 days post-infection. Transcript levels were normalized by the levels of the reference gene *EF1-α*. The error bars represent the SD of three biological replicates. Asterisks indicate statistical significant differences in a *t*-test with *p* < 0.05 comparing the values of each tissue with the control YEM root at the same time point.

To determine whether *NIPK* is expressed in *P. vulgaris* roots and nodules, we quantified *NIPK* mRNA levels by reverse transcription followed by quantitative PCR (RT-qPCR). *NIPK* transcripts showed a higher accumulation in inoculated roots at 4 and 7 days post-inoculation (dpi) as compared with mock-inoculated roots. In contrast, levels of *NIPK* were lower in 10 dpi nodules than in inoculated roots, and expressed at very low levels in nodules of 14 and 21 dpi (Figure 4). An inspection of expression data at the *P. vulgaris* Gene Atlas database^3^ also showed that the highest level of *NIPK* transcripts was detected in roots and the lowest in nodules, whereas it was intermediate in leaves (Supplementary Figure S4A). In addition, *NIPK* mRNA levels were 3.5-fold lower in denodulated roots compared to control fertilized roots collected 21 dpi.

Considering that *NF-YC1* levels are induced in response to the highly efficient strain SC15 at 24 h post inoculation (hpi; Zanetti et al., 2010; Rípodas et al., 2015), we asked whether *NIPK* expression showed a similar pattern. To answer this, we analyzed RNA-seq data from *P. vulgaris* roots inoculated with different *R. etli* strains in the Mesoamerican accession NAG12 and the Andean accession Alubia at 24 hpi (Clúa et al., 2022). The results indicate that *NIPK* mRNA accumulation remained constant upon inoculation with strains that carry either *nod*C-α (SC15 and CE3) or *nod*C-δ (55 N1 and 124 N1) alleles relative to the mock-inoculated control in both accessions (Supplementary Figure S4B). Taken together, expression data suggest that *NIPK* transcripts are expressed in roots and its levels increase during nodule primordial formation.

### Nodule organogenesis and development is affected in *NIPK* RNAi roots

To functionally characterize the role of *NIPK* in *P. vulgaris* roots, we generated transgenic hairy roots expressing two specific RNA interferences (RNAi) complementary to the 3′ untranslated region (UTR) of *NIPK* (*NIPK* RNAi 1) or to the kinase domain (*NIPK* RNAi 2; Supplementary Figure S5A). The vector chosen for the expression of the RNAi also contained the coding sequence of *gfp* under the control of the *rolD* promoter, which allowed the identification of transgenic roots for the phenotypic analysis (Supplementary Figure S5B). Hairy roots transformed with *NIPK* RNAi 1 or 2 showed 79% and 95% of *NIPK* silencing, respectively, compared to *GUS* RNAi roots. To test the specificity of the silencing, we quantified the transcript levels of the *Phvul011G070500* gene, which encodes the closest homolog of NIPK with a similar protein length and domain organization. The results showed that *Phvul011G07500* was strongly silenced in *NIPK* RNAi 2 roots, whereas it was not affected by *NIPK* RNAi 1 expression (Supplementary Figure S5C).

**Figure 5:**
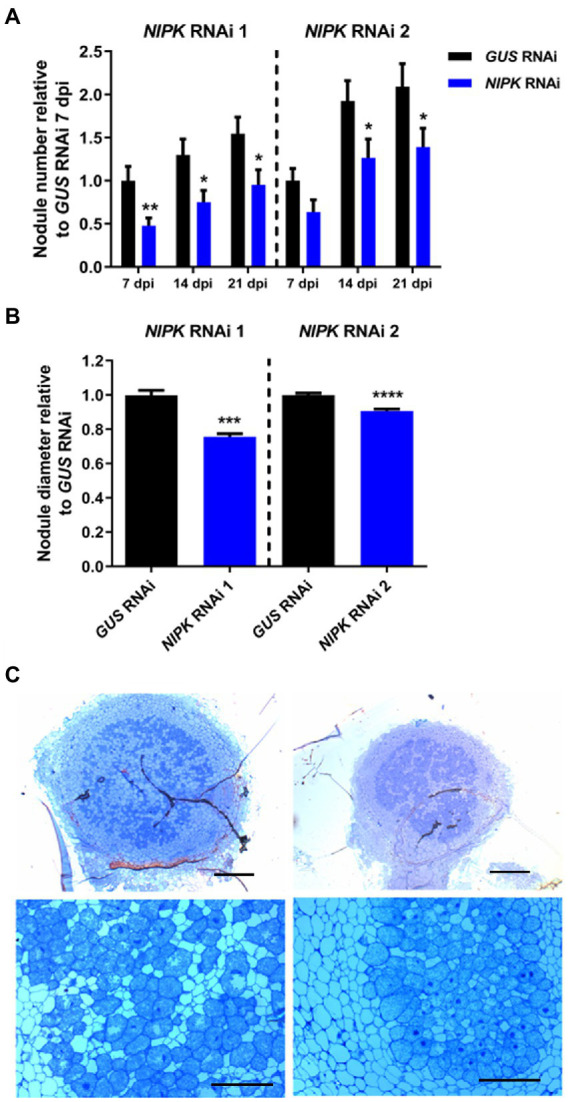
*NIPK* silencing affects nodulation. Transgenic roots expressing *NIPK* RNAi or *GUS* RNAi were grown for 7 days without nitrate and inoculated with the SC15 strain of *Rhizobium etli*. **(A)** The number of nodules was recorded at 7, 14, and 21 days after infection. The *GUS* RNAi value at 7 days post-infection (dpi) is used as reference. **(B)** Nodule diameter was measured at 21 dpi. Error bars in **(A,B)** represent SEM. Significant differences in a *t*-test are indicated with asterisks (^*^, *p* < 0.05; ^**^, *p* < 0.01; ^***^, *p* < 0.001; ^****^, *p* < 0.0001). **(C)** Nodule structure of *GUS* RNAi (left panels) or *NIPK* RNAi 1 (right panels) plants 14 dpi with *R. etli* stained with toluidine blue. Scale bars: 200 μm (upper panels), 50 μm (lower panels).

Considering that NIPK was identified as an interactor of NF-YC1, we assessed the effect of posttranscriptional silencing on the symbiotic interaction. Transgenic hairy roots expressing *NIPK* RNAi 1 or 2 were subjected to a time-course nodulation experiment with the cognate strain *R. etli* SC15. *NIPK* RNAi 1 and 2 roots developed fewer nodules than control roots expressing *GUS* RNAi at all time points recorded (Figure 5A; Supplementary Figure S6A). In addition to the reduction of the nodule number, *NIPK* RNAi 1 and 2 nodules showed significant reductions in their diameter of 24 and 9%, respectively (Figure 5B; Supplementary Figure S6B). Optical microscopy of semi-thin sections of 21 dpi nodules revealed that *NIPK* RNAi 1 nodules are infected with rhizobia and that the size of the infected area is similar to control nodules (Figure 5C). The morphology and size of the cells in the central zone of the *NIPK* RNAi 1 nodules were also similar to that of *GUS* RNAi nodules (Figure 5C, lower panels).Altogether, these results suggest a role of *NIPK* in nodule formation and development, resembling what was observed for *NF-YC1*.

### *NIPK* is required for IT formation, but not for its progression

**Figure 6:**
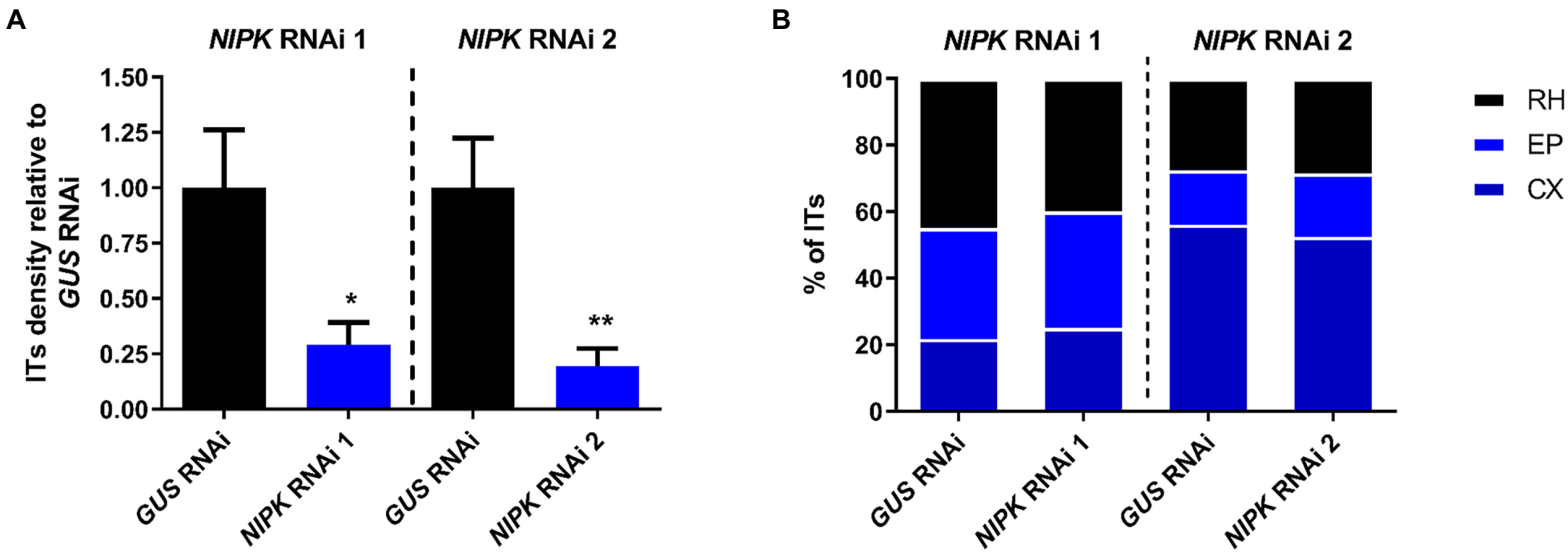
*NIPK* silencing affects formation, but not progression of ITs. *GUS* or *NIPK* RNAi composite plants were inoculated with *Rhizobium etli* CFN5X expressing DsRED. **(A)** ITs were visualized and quantified at 4 dpi and normalized by root length. Error bars represent SEM. Significant differences in a *t*-test are indicated with asterisks (^*^, *p* < 0.05; ^**^, *p* < 0.01). **(B)** ITs were classified in three categories: RH, when just growth inside the root hair; EP when growth to epidermal cells (ending in the base of the trichoblast or in an adjacent epidermal cell); and CX for these reaching cortical cells. Percentages of ITs in each category are presented.

Since *NIPK* RNAi roots showed a reduced number of nodules as compared with control roots, we asked whether the infection was also compromised. To answer this, we inoculated *NIPK* RNAi and *GUS* RNAi roots with a *R. etli* strain that constitutively expresses the red fluorescence protein (dsRed), allowing to follow the infection events by fluorescence microscopy. Four days after infection, *NIPK* RNAi 1 and 2 roots showed a significant reduction in the number of ITs per centimeter of root as compared with control roots (Figure 6A; Supplementary Figure S7A). To determinate if the progression of ITs was affected, we classified them in three categories: ITs ending in the root hair, ITs ending in epidermal cells, or ITs reaching cortical cell, as previously reported (Zanetti et al., 2010). The results show that IT progression was not significantly affected in *NIPK* RNAi roots (Figure 6B; Supplementary Figure S7B). In conclusion, *NIPK* seems to be required for initiation of ITs, but not for their elongation/progression toward the nodule primordium.

### *NIPK* is necessary for induction of cell cycle and early nodulin genes

**Figure 7:**
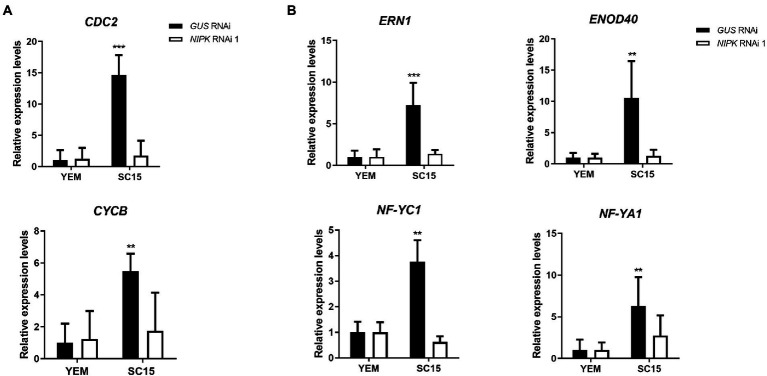
Expression analysis of cell cycle genes and early symbiotic genes. *GUS* or *NIPK* RNAi roots were inoculated with *Rhizobium etli* SC15 or mock-inoculated (YEM) and collected 24 h after inoculation. Levels of *cyclin B* (*CYCB*) and *CDC2* **(A)** or the symbiotic genes *ERN1*, *ENOD40*, *NF-YC1,* and *NF-YA1* **(B)** were quantified by qRT-PCR, normalized by *EF1-α* levels, and expressed relative to the *GUS* RNAi YEM control. Double and triple asterisks indicate that expression values in *NIPK* RNAi 1 are significantly different from those in *GUS* RNAi roots in an unpaired two-tailed *t*-test with *p* < 0.01 and *p* < 0.001, respectively. Error bars represent the SD of three independent experiments.

As previously mentioned, NF-Y transcription complexes regulate the activation of cell cycle genes. In order to test the possible implication of *NIPK* in the regulation of G2/M transition genes, we measured the expression of *Cyclin B* (CYCB) and *CDC2* mRNA levels in *NIPK* and *GUS* RNAi roots. The increase of both transcripts in response to rhizobia inoculation was impaired in plants with reduced *NIPK* levels (Figure 7A), suggesting this protein pseudokinase is required for the induction of cell cycle genes that are activated during nodule primordia formation.

Considering the effect of *NIPK* post-transcriptional gene silencing on infection and nodule formation, we evaluated whether the expression of early nodulation genes was affected by silencing of *NIPK*. As observed in Figure 7B, the induction of the ethylene response factor required for nodulation *ERN1* (Middleton et al., 2007; Cerri et al., 2012) and Early Nodulin 40 (*ENOD40*; Crespi et al., 1994) was impaired in roots that express *NIPK* RNAi 1. Moreover, accumulation of two of the three components of the NF-Y complex, *NF-YC1* and *NF-YA1,* was also impaired in *NIPK* RNAi roots. These results suggest that *NIPK* is necessary for the activation of early symbiotic genes that participate of rhizobial infection and nodule organogenesis.

### *NIPK* RNAi does not affect strain preference

**Figure 8:**
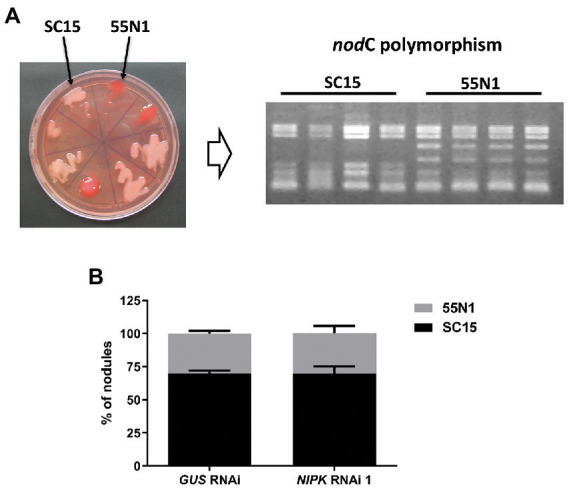
*NIPK* RNAi does not affect strain preference. **(A)** Representative plate showing the *nod*C-α strain SC15 (pale pink) and the *nod*C-δ strain 55 N1 (red) of *Rhizobium etli* growing in Congo red media. Identity of nodule occupying strains was confirmed by ARFLP analysis of the *nodC* polymorphism. **(B)** Percentage of nodule occupancy by each strain in *GUS* and *NIPK* RNAi 1. Error bars represent SEM of two independent experiments.

Considering that *NF-YC1* is involved in partner selection between *nod*C-α and *nod*C-δ *R. etli* strains, we hypothesized that NIPK could be required for the regulation of this preference through its interaction with NF-YC1. To answer this, both *NIPK* RNAi 1 and *GUS* RNAi roots were co-inoculated with an equicellular mixture of SC15 (*nod*C-α) and 55 N1 (*nod*C-δ) strains. Nodules were collected at 21 dpi and rhizobia within each nodule were isolated and identified as *nod*C-α (SC15) or *nod*C-δ (55 N1) strains based on the *nod*C polymorphism (Figure 8A). As previously reported, nodules were preferentially occupied by the *nod*C-α strain in *GUS* RNAi (Aguilar et al., 2004; Zanetti et al., 2010). Reduction of *NIPK* levels by RNAi 1 did not affect the occupancy of nodules by these two different strains of *R. etli* (Figure 8B), indicating that unlike *NF-YC1*, *NIPK* is not required for the selection of rhizobial strains that will occupy the nodule.

## Discussion

In this work, we report the identification and characterization of a protein kinase that interacts with NF-YC1 to control rhizobial infection and nodule organogenesis. Based on the sequence analysis, NIPK would be a pseudokinase without phosphotransferase activity, since two of the three amino acids predicted to be necessary for the catalytic activity are not conserved in the NIPK protein sequence (Hanks, 2003; Boudeau et al., 2006). However, this criterion is not absolute, and many proteins classified as pseudokinases were shown to be catalytically active (Xu et al., 2000; Abe et al., 2001; Min et al., 2004). The protein kinases that do not have all the features defined as necessary for kinase activity account for approximately 10% of all protein kinases (Boudeau et al., 2006); however, many of them play important cellular roles mediating the interaction and regulation of active protein kinases (Llompart et al., 2003; Chevalier et al., 2005; Castells and Casacuberta, 2007; Halter et al., 2014; Kumar et al., 2017). For example, the perception of the Nod factor in nitrogen-fixing symbiosis is mediated by two LysM ectodomain containing receptor-like kinases, one of which has a dead kinase intracellular domain (Radutoiu et al., 2003, 2007; Smit et al., 2007). Independently of the putative kinase activity, NIPK seems to have a conserved function in different plants since all the three amino acids that interact with ATP and Mg^2+^ to achieve proton transfer are conserved in its putative orthologs from other species, from lycophytes to angiosperms.

According to the classification of pseudokinases, NIPK belongs to the F group, where the lysine in VAIK (domain II) and the aspartic acid in HRD (domain VIb) are not conserved (Boudeau et al., 2006). The best homologs of NIPK in *Drosophila melanogaster* and human (*Homo sapiens*) are Pelle and IL-1 receptor-associated kinase (IRAK) 4, respectively. Both proteins have catalytic activity, but IRAK4 is closely related to IRAK2, a pseudokinase. Pelle and IRAK proteins participate in the responses triggered by Toll receptors. Interestingly, Pelle participates of the nuclear translocation of the transcription factor dorsal, which establishes the dorsoventral polarity of fly embryos (Shelton and Wasserman, 1993). These examples show that kinases and pseudokinases can participate in transcription factor translocation to the nucleus in response to external stimuli.

### Subcellular localization of NF-YC1 and NIPK

As expected by the TM domain present in the protein, NIPK localizes to the plasma membrane. In addition, NIPK was also detected in the cytoplasm, where physically interacts with NF-YC1 (Figure 3). This fraction of NIPK located in the cytoplasm could be responsible for the biological function of NIPK in its association with NF-YC1. Considering that NIPK acts a positive regulator of NF-YC1, its function could be associated with the translocation of the dimer NF-YB/NF-YC to the nucleus to activate the target genes of the NF-Y trimer and other transcriptional complexes that include NF-YC1. However, overexpression of *NIPK* was not enough to affect the subcellular localization of NF-YC1 (Supplementary Figure S3), suggesting that another factor could be limiting the regulation of NF-YC1 translocation to the nucleus. Regulation by phosphorylation has been reported for NF-Y complexes in human cells, where the NF-YA subunit is phosphorylated in two serine residues by the cyclin-dependent kinase CDK2. A mutant version of NF-YA where these serine amino acids are substituted was able to form the trimer with NF-YB and NF-YC, but the DNA binding and the activation of the target genes *CDC2* and *CDK2* were inhibited (Yun et al., 2003; Chae et al., 2004; Chan et al., 2010; Dolfini et al., 2012). It was also reported that NF-YA and NF-YB can be acetylated to prevent their degradation *via* the proteasome (Currie, 1998; Li et al., 1998; Manni et al., 2008; Dolfini et al., 2012). Considering that pseudokinases can function as scaffolds to form multiprotein complexes, it is possible that NIPK would participate in phosphorylation, acetylation, or any other posttranslational modification that affects the activity of NF-YC1.

### Role of NIPK in symbiosis

Functional analyses suggest that NIPK is a positive regulator of the initiation of bacterial infection and nodule organogenesis, since transgenic roots expressing a *NIPK* RNAi showed a reduction of IT density, as well as a lower number of nodules as compared with *GUS* RNAi control roots. In addition, *NIPK* seems to be necessary for activation of cell cycle genes that are crucial to reactivate cell division of cortical cells that will form the nodule primordia. This phenotype is similar to *NF-YC1* silenced plants, which also showed defects in rhizobial infection and nodule development (Zanetti et al., 2010). This similarity between both phenotypes suggests that NIPK could be involved in the modulation or relocalization of NF-YC1 during the root nodule symbiosis. However, reduction of *NIPK* levels does not affect IT progression or the strain preference observed in common bean when *nodC*-α and *nodC*-δ strains are included in co-inoculation assays, indicating that these functions of NF-YC1 do not require the participation of NIPK.

On the other hand, similarly to that observed for *NF-YC1* and *NF-YA1*, *NIPK* is required for activation of cell cycle genes *CYCB* and *CDC2* upon rhizobia infection. Since phosphorylation and acetylation of NF-Y subunits are required for full activity of the NF-Y complex in mammals (Dolfini et al., 2012), the lack of activation of the *CYCB* and *CDC2* cell cycle genes observed in *P. vulgaris NIPK* RNAi roots at early stages of the root nodule symbiosis might be related the lack of post-translational modification or translocation to the nucleus of the NF-Y subunits.

Accumulation of early symbiotic gene transcripts is also abolished in *NIPK* RNAi plants, indicating that NIPK participates in the molecular responses leading to activation of the genetic programs associated to rhizobial infection and nodule organogenesis. This result is in agreement with the observed phenotype, where IT formation is strongly reduced in *NIPK* RNAi roots.

In summary, functional analysis revealed the role of a pseudokinase in infection and nodule organogenesis, adding a new player in the regulation of NF-Y complexes in the context of root nodule symbiosis.

## Data availability statement

The original contributions presented in the study are included in the article/Supplementary material, further inquiries can be directed to the corresponding author.

## Author contributions

MZ and FB planned and designed the research. JC, CRí, CRo, and MB performed the experiments. JC, CRí, CRo, MB, MZ, and FB analyzed and discussed the data. FB wrote the manuscript. All authors contributed to the article and approved the submitted version.

## Funding

This work was financially supported by grants from ANPCyT, Argentina (PICT 2017/00069, PICT 2019/00029, and PICT2020-00053). All authors are funded by CONICET.

## Conflict of interest

The authors declare that the research was conducted in the absence of any commercial or financial relationships that could be construed as a potential conflict of interest.

## Publisher’s note

All claims expressed in this article are solely those of the authors and do not necessarily represent those of their affiliated organizations, or those of the publisher, the editors and the reviewers. Any product that may be evaluated in this article, or claim that may be made by its manufacturer, is not guaranteed or endorsed by the publisher.

## Footnotes

^1^ http://www.cbs.dtu.dk/services/TMHMM/

^2^ http://www.uniprot.org/

^3^ https://www.zhaolab.org/PvGEA/
