# Supplementary material for: NIPK, a protein pseudokinase that interacts with the C subunit of the transcription factor NF-Y, is involved in rhizobial infection and nodule organogenesis
Source: Front Plant Sci. 2022 Sep 21;13:992543. doi: 10.3389/fpls.2022.992543 (PMC9532615; doi:10.3389/fpls.2022.992543)
Supplement: Supplementary file 1 [file Table_1.DOC]

**Table S1.** Primers used in this study

| Primer | Sequence |
| --- | --- |
| His-FLAG Fw | CACCATGGGACATCACCATCATCACC |
| NF-YC1 ORF Rv | CTAATGTTCTGGGGATGGTTGACG |
| NIPK ORF Fw | CACCATGTTGATACTAGTTCTTGGACTAGC |
| NIPK ORF no ATG Fw | CACCTTGATACTAGTTCTTGGACTAGCTTC |
| NIPK ORF Rv | CTAGAACATAATTTCTTCAAGTTTCTTGAGG |
| NIPK ORF no STOP Rv | GAACATAATTTCTTCAAGTTTCTTGAGGAC |
| ∆NIPK Fw | CACCGTGATTGGAAAATCCAACTATGGAAC |
| NIPK RNAi 1 Fw | CACCGTGAGCTTGTGACATGGACAATG |
| NIPK RNAi 1 Rv | GCTACAAATTCTTGGACAGCAGCAC |
| NIPK RNAi 2 Fw | CACCGAAGCTTCTTGTTCATCCCTTC |
| NIPK RNAi 2 Rv | TTACAAGGCACCTGAGCTCA |
| qNIPK Fw | CACCGTGAGCTTGTGACATGGACAATG |
| qNIPK Rv | GCTACAAATTCTTGGACAGCAGCAC |
| qPhvul.011G070500 Fw | GTGGTCCGTTGTAGTTGCCA |
| qPhvul.011G070500 Rv | TGAAAGGGCAAGAGGCAAAAC |
| qEF1-α Fw | AACTACCACCGGCCACTTGAT |
| qEF1-α Rv | AGCACCCAGGCATACTTGAAT |
| qERN1 Fw | CTTCTCGAATTCGGAATCTT |
| qERN1 Rv | TGTTGCCATTGCCATTGTT |
| qENOD40 Fw | AGTTTTGTTGGCAAGCATCC |
| qENOD40 Rv | TAAGCGCAAGCAAACTGTTG |
| qNF-YA1 Fw | TCATTTTGGATCTTCTGTGCACAC |
| qNF-YA1 Rv | GCTTCTTCATCTGGTCTCATAAAGG |
| qNF-YC1 Fw | GCAGGGCTATATCGGTCTTTTC |
| qNF-YC1 Rv | GAGTAACATTTTGGATTCAATG |
| qCycB Fw | TTCTTGTGAGTAACCCGTGTG |
| qCycB Rv | CATGTCCCATTTTCTATCTGAGTTG |
| qCdc2 Fw | CAGCAAAAGAATTACCGCCAGGAT |
| qCdc2 Rv | GCACGCATTTCTCGCATAGTCAA |
| nodC Fw | TGCTTGACACAACCAGCACTG |
| nodC Rv | GACAGCCAGTCGCTATTGGTCA |
| p35S BiFC Fw | CACTATCCTTCGCAAGACCC |
| p35S HF Fw | GAGGACACGCTCGAGTATAAGAGC |
| OCS Rv | CATGCGATCATAGGCGTCTCG |
