# Supplementary material for: NIPK, a protein pseudokinase that interacts with the C subunit of the transcription factor NF-Y, is involved in rhizobial infection and nodule organogenesis
Source: Front Plant Sci. 2022 Sep 21;13:992543. doi: 10.3389/fpls.2022.992543 (PMC9532615; doi:10.3389/fpls.2022.992543)
Supplement: Supplementary file 2 [file Data_Sheet_1.PDF]

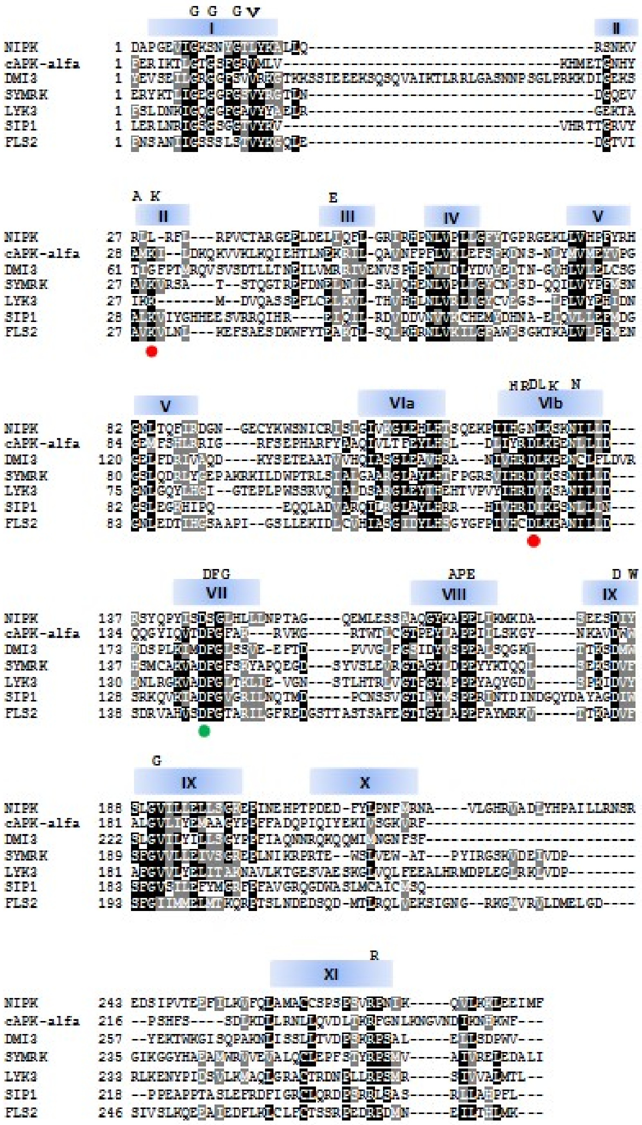

Figure S1. Conservation of kinase subdomains in the NIPK catalytic domain.

Multiple sequence alignment of the protein kinase domain of NIPK with those of other protein kinases reported to be catalytically active: DMI3 (Gleason et al., 2006), SYMRK (Yoshida and Parniske, 2005), LIK3 (Jayaraman et al., 2017), SIP2 (Chen et al., 2012), FLS2 (Lu et al., 2010) and cPKA-alfa. The alignment was generated using Clustal Omega. Conserved regions were colored using the BOXSHADE server. Identical amino acids are highlighted in black, and similar residues are highlighted in gray boxes. The eleven characteristic subdomains of the protein kinase family are indicated by light blue boxes with roman numbers. The consensus amino acids of the different subdomains are indicated in bold above the boxes. The colored circles indicate the positions of the three amino acids required for the phosphotransfer reaction (K, D and D). The green or red color of the circles indicates whether these amino acids are conserved or not in NIPK, respectively.

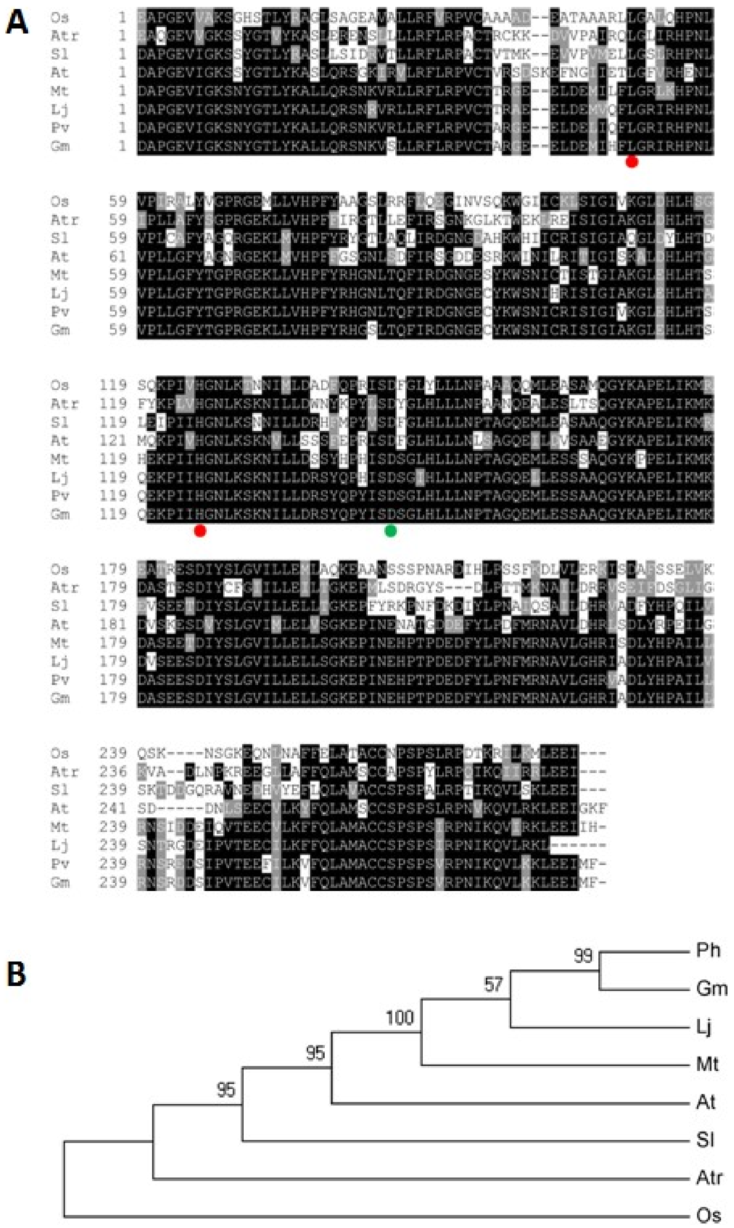

Figure S2. (**A**) Alignment of the best homologs of NIPK from different species. The alignment shows the conservation of key amino acids in the motives VAIK (II subdomain), HRD (VIb subdomain) and DFG (VII subdomain). Putative orthologs were identified in *Medicago truncatula* (Mt), *Arabidopsis thaliana* (At), *Lotus japonicus* (Lj), *Glycine max* (Gm), *Amborella trichopoda* (Atr), *Solanum lycopersicum* (Sl) and *Oryza sativa* (Os). The colored circles indicate whether each of the three amino acids required for the phosphotransfer reaction are conserved (green) or not (red) compared with the amino acids found in canonical kinases. (**B**). Phylogenetic tree of NIPK and the putative orthologs used in the alignment. The phylogenetic tree was generated using MEGA7. Numbers represent bootstrap values obtained from 1,000 trials.

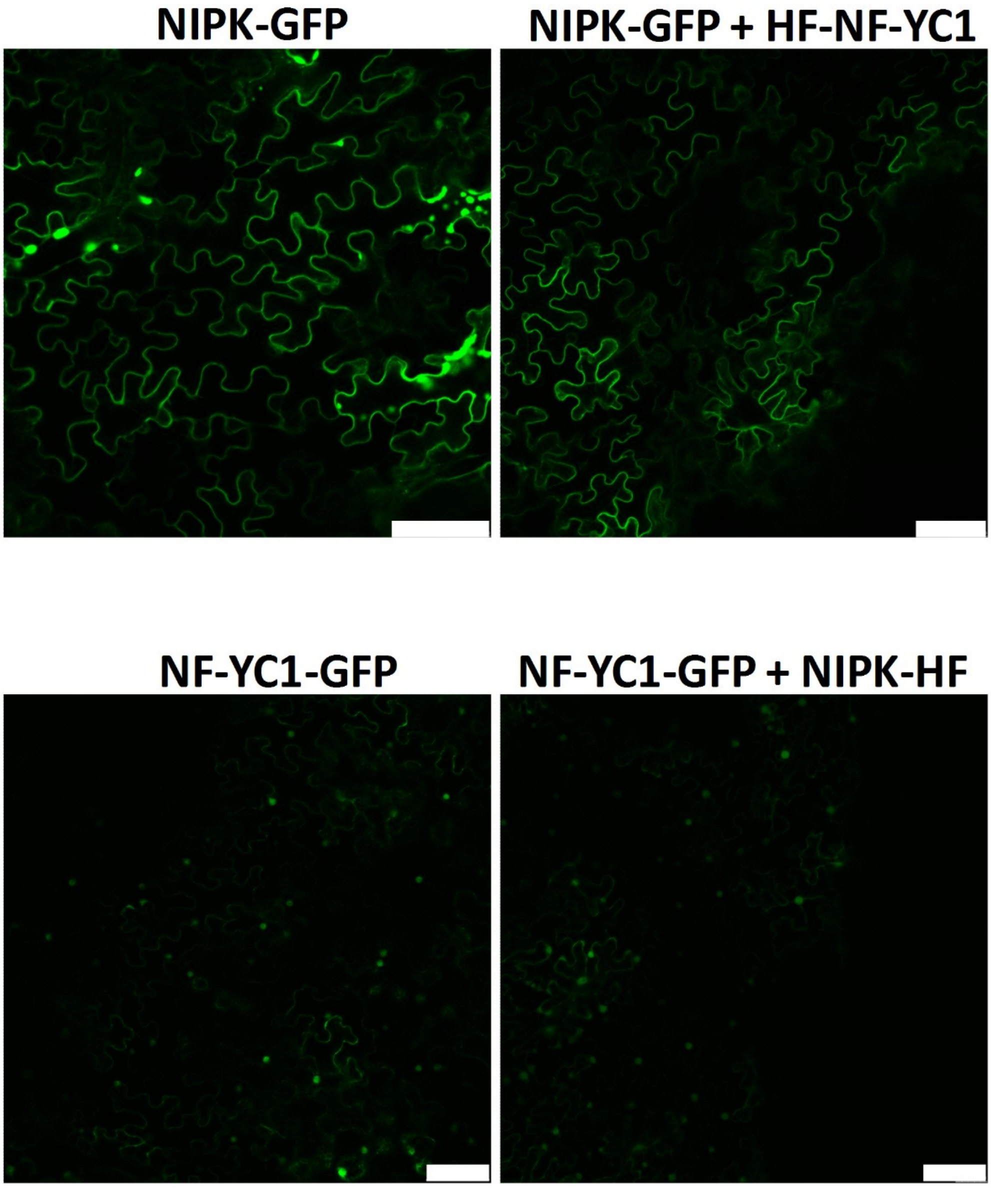

Figure S3. Subcellular localization of NIPK is not affected by overexpression of NF-YC1 and *vice versa*.
Upper panels: *N. benthamiana* epidermal leaf cells expressing NIPK-GFP alone (left panel) or together with HF-NF-YC1 (right panel). Lower panels: *N. benthamiana* epidermal leaf cells expressing NF-YC1-GFP alone (right panel) or with NIPK-HF. Confocal laser microscopy images of the GFP fluorescence are shown. Scale bars: 50 µm.

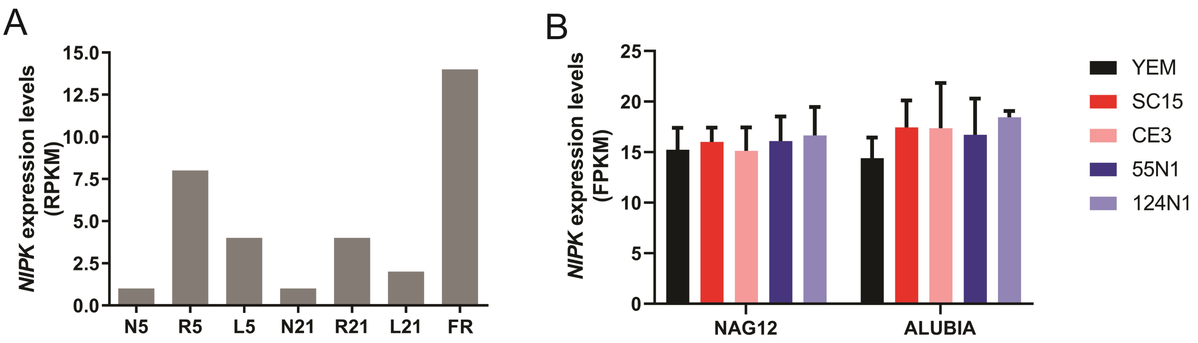

Figure S4. Expression levels of *NIPK* in different tissues.
(**A**) Data were obtained from the *P. vulgaris* Gene Atlas. N5, R5, L5, N21, R21 and L21 indicate nodule (N), root (R) or leaf (L) tissue from 5 or 21 days post infection (dpi). FR corresponds to root tissue from fertilized plants collected at 21 dpi. (**B**) Expression of *NIPK* in roots of NAG12 (Mesoamerican accession) or Alubia (Andean accession) common bean plants 24 hpi with strains of *R. etli* carrying the *nodC*-α (SC15 and CE3) or the *nodC*-δ (55N1 and 124N1) allele of the *nodC* gene. YEM corresponds to the control mock-inoculated plants. Data were obtained from Clúa et al., 2022. FPKM: fragments per kilobase per million.

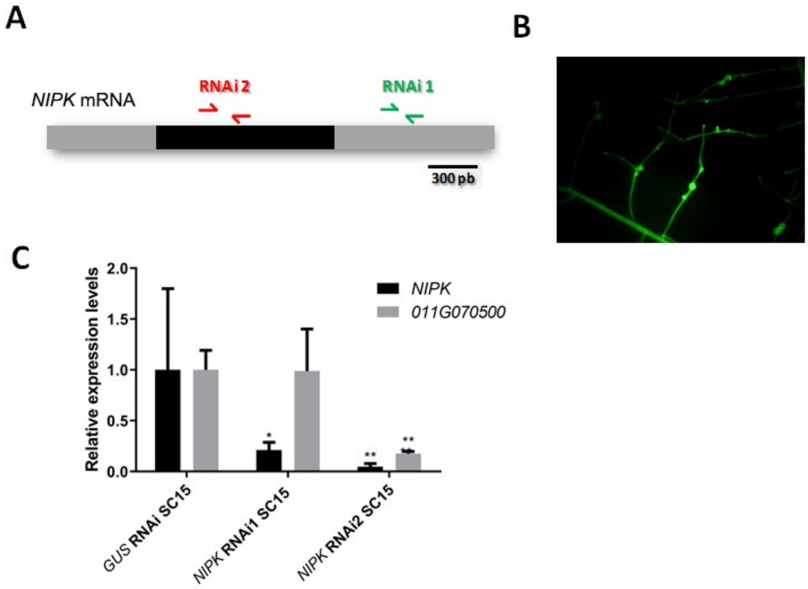

Figure S5. Post-transcriptional silencing of *NIPK*.
(**A**) Schematic representation of the *NIPK* mRNA showing untranslated sequences (gray), the coding sequence (black) and the position of the primers used for construction of RNAi 1 (green) and 2 (red). (**B**) Representative *NIPK* RNAi transgenic roots expressing GFP. (**C**) Expression levels of *NIPK* and *Phvul.011G070500* in inoculated transgenic roots expressing *GUS*, *NIPK* RNAi 1 and *NIPK* RNAi 2. Significant differences in a t-test are indicated with asterisks (*, $p<0.05$; **, $p<0.01$). Transcript levels were normalized by the levels of the reference gene *EF-1α*.

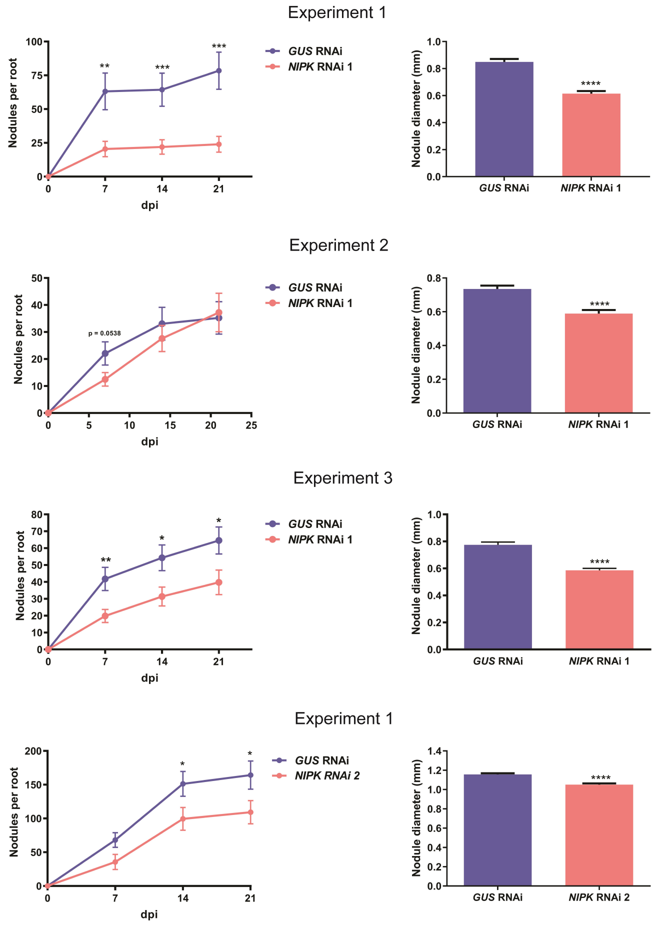

Figure S6. Biological replicates of the experiments shown in Figure 5.
Transgenic roots expressing *NIPK* RNAi or *GUS* RNAi were grown for 7 days without nitrate and inoculated with the SC15 strain of *R. etli*. The number of nodules was recorded at 7, 14 and 21 days after infection and normalized by the *GUS* RNAi value at 7 days post-infection (dpi). Nodule diameter was measured at 21 dpi. Error bars represent SEM. Significant differences in a t-test are indicated with asterisks (*, $p<0.05$; **, $p<0.01$; ***, $p<0.001$; ****, $p<0.0001$).

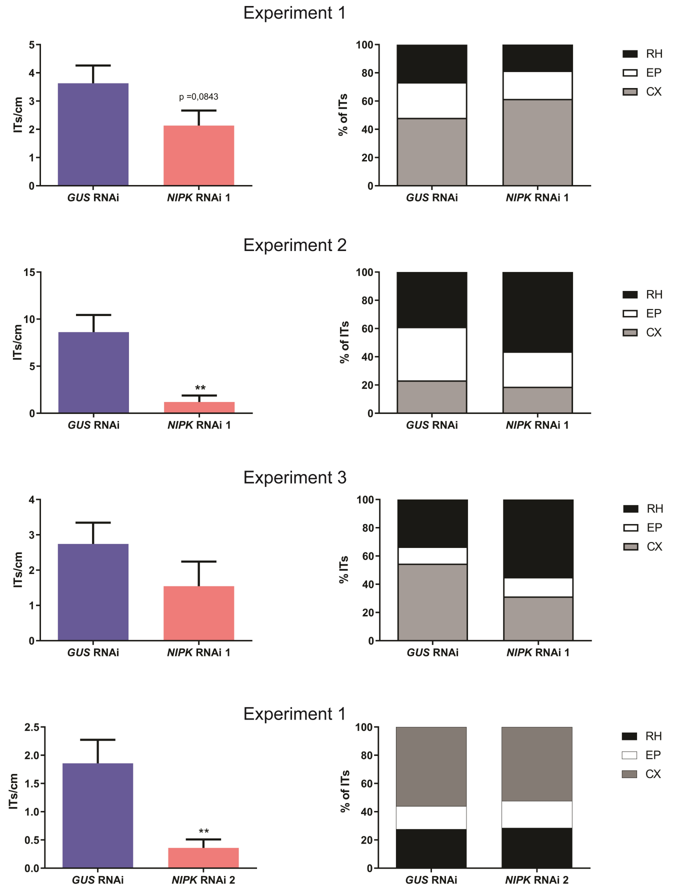

Figure S7. Biological replicates of the experiments shown in Figure 6.
GUS or *NIPK* RNAi composite plants were inoculated with *R. etli* CFN5X expressing DsRED. (**A**) ITs were visualized and quantified at 4 dpi and normalized by root length. Error bars represent SEM. Significant differences in a t-test are indicated with asterisks (**, $p<0.01$). (**B**) ITs were classified in three categories: RH, when just growth inside the root hair; EP when growth to epidermal cells (ending in the base of the trichoblast or in an adjacent epidermal cell); and CX for these reaching cortical cells. Percentages of ITs in each category are presented.
